# Prevalence of COVID-19-Associated Pulmonary Aspergillosis: Critical Review and Conclusions

**DOI:** 10.3390/jof8040390

**Published:** 2022-04-12

**Authors:** Matthias Egger, Linda Bussini, Martin Hoenigl, Michele Bartoletti

**Affiliations:** 1 Department of Internal Medicine, Division of Infectious Diseases, Medical University of Graz, 8036 Graz, Austria; m.egger.med@hotmail.com; 2 Biotechmed-Graz, 8036 Graz, Austria; 3 Infectious Diseases Unit, IRCCS Azienda Ospedaliero-Universitaria di Bologna, Policlinico di Sant’Orsola, 40138 Bologna, Italy; linda.bussini@gmail.com (L.B.); m.bartoletti@unibo.it (M.B.); 4 Clinical and Translational Fungal—Working Group, University of California San Diego, La Jolla, CA 92093, USA; 5 Department of Medicine, Division of Infectious Diseases and Global Public Health, University of California San Diego, San Diego, CA 92093, USA; 6 Department of Medical and Surgical Sciences, Alma Mater Studiorum University of Bologna, 40126 Bologna, Italy

**Keywords:** invasive aspergillosis, COVID-19, serum, bronchoalveolar lavage

## Abstract

First reports of cases and case series of COVID-19-associated pulmonary aspergillosis (CAPA) emerged during the first months of the pandemic. Prevalence rates varied widely due to the fact that CAPA was, and still remains, challenging to diagnose in patients with COVID-19-associated acute respiratory failure (ARF). The clinical picture and radiological findings of CAPA are unspecific and can resemble those of severe COVID-19. Hence, mycological evidence became a key component in establishing a diagnosis. However, blood tests lack sensitivity in early treatable phases of CAPA and once positive, mortality has been shown to exceed 80% despite systemic antifungal therapy. The primarily airway invasive growth in non-neutropenic patients and the late occurrence of angioinvasion in the course of disease may mainly account for these diagnostic obstacles. Testing of bronchoalveolar lavage (BAL) is therefore crucial in the diagnostic process, but was rarely performed during the early phase of the pandemic, which potentially interfered with the accuracy of reported prevalence. Current guidelines recommend treatment of CAPA during its early airway invasive phase, which may result in some overtreatment (i.e., treatment in patients that may not develop angioinvasive infection) and adverse drug events, yet there is no viable alternative approach. Timely treatment of cases needs to be ensured for patients with mycological evidence of CAPA in the lower respiratory tract given the independent contribution of CAPA to devastating mortality rates of around 50% that have been shown in multiple studies. Here, we review the evolution of reported CAPA prevalence and the role of CAPA as an important opportunistic infection affecting COVID-19 patients in intensive care units (ICUs).

## 1. Introduction

COVID-19-associated pulmonary aspergillosis (CAPA) is an opportunistic secondary infection, primarily affecting patients in the intensive care unit (ICU) with COVID-19 acute respiratory failure (ARF). Patients affected by COVID-19 ARF and CAPA exhibited increased mortality compared to patients with COVID-19 ARF but without CAPA [1,2,3]. Besides clinical factors, including invasive respiratory support, concomitant corticosteroid/anti-interleukin-6 treatment, and older ager, immunologic mechanisms, which accompany COVID-19 infection, have been shown to be independent risk factors resulting in an increased susceptibility to invasive fungal infections [2,3,4]. In the absence of consensus definitions for CAPA, classification criteria and CAPA definitions varied widely early in the pandemic, resulting in a wide range of reported CAPA prevalence rates between 1.6% and 38% in patients with COVID-19 ARF requiring invasive ventilation [5,6,7,8,9,10]. While application of the European Confederation of Medical Mycology (ECMM)/International Society for Human and Animal Mycoses (ISHAM) consensus criteria resulted in a significant reduction of CAPA prevalence [11], recent studies continue to report varying numbers between ICUs. Potential explanations for these findings may be ongoing regional differences in (1) therapeutic approaches to COVID-19, (2) diagnostic aggressiveness and accuracy, (3) genetic predisposing factors and (4) burden of *Aspergillus* exposure. The wide range of CAPA prevalence rates observed in ICUs has also sparked a scientific debate on whether or not CAPA has been overemphasized in the literature. Here we will review pros and cons of why CAPA may be overemphasized, dissect up to date evidence to highlight strengths and weaknesses, and last but not least highlight some open questions that are still waiting to be answered.

## 2. Early Overestimation of CAPA Rates in the Literature and the Impact of ECMM/ISHAM Consensus Definitions

Studies published before September 2020 reporting on invasive pulmonary aspergillosis complicating the course of disease in ICU patients with COVID-19 associated ARF, yielded highly varying prevalence rates [7,12,13] affecting an average of 3.1% (range 0.7–7.7%) [10,14,15,16,17,18] of patients hospitalized with COVID-19, 8.9% (range 2.5–39%) of patients admitted to the ICU [5,6,13,16,19,20,21,22,23,24] and an average of 20.1% (range 1.6–38%) requiring invasive ventilation [5,6,7,8,9,10]. In the absence of uniform diagnostic criteria, these important early studies naturally used a wide range of mycological criteria to classify patients with CAPA. To avoid aerosol exposure and transmission there was also a reluctance to perform bronchoscopies and many of these studies relied on unspecific mycological evidence, such as culture growth or non-validated galactomannan (GM) detection in tracheal aspirate (TA) [19] or beta-D-glucan testing from serum, which is rather a general prognostic marker in the ICU than CAPA specific [25], as mycological evidence for putative CAPA. These circumstance, combined with the unspecific clinical and radiological presentation of CAPA in patients with COVID-19 ARF, probably led to overestimation and the high variability observed.

The application of the more conservative ECMM/ISHAM consensus criteria for CAPA published in late 2020 has resulted in a significant reduction of reported prevalence rates to about half (i.e., reduction of the mean incidence of probable/proven CAPA cases from 19% to 11.9% in one review) of what has been reported before when primarily modified Blot criteria were used, bringing the prevalence of CAPA cases closer to that suggested by autopsy studies [2,3,26,27]. Better applicable criteria for CAPA management were needed urgently and in a timely manner to allow for comparison of prevalence rates between studies and a more accurate estimation of disease frequency. Thus, these criteria partly had to rely on expert opinions and were made based on limited data that was available at that time. Fifteen months after the publication and implementation of the new criteria, their substantial benefit in CAPA management has undoubtedly been shown. Still, there is also room for improvement, which needs to be considered in order to warrant their optimal utilization. On a global scale, bronchoscopy is scarcely available in low/middle income countries (LMIC), yet, if no lower respiratory tract specimens can be obtained (i.e., BAL, non-bronchial lavage (NBL)) [28,29], application of ECMM/ISHAM criteria is not possible for cases diagnosed early. To overcome this limiting circumstance, inclusion of less specific mycological evidence (e.g., combination of upper respiratory tract specimens like TA and sputum) could be considered as weak mycological criteria that could be applied in combination only, which would allow for classification of possible disease. While diagnostic tools (e.g., GM testing, PCR, culture) and their respective cut-offs have only been rarely evaluated for these materials [19,30], feasibility for low and middle income countries (LMICs) to implement criteria needs to be provided in order to achieve worldwide applicability. This urgent need is further highlighted by the devastating mortality rates coming along with GM detection in blood during the course of the disease [26]. Another aspect which needs to be considered is the question whether a single slightly positive serum GM and/or serum LFA result, in the absence of any other mycological evidence, is sufficient evidence for classification of probable CAPA [26,30]. This could result in higher prevalence rates, which once more come along with overtreatment and associated detriments. Lastly, classification of possible CAPA by investigation of non-bronchoscopic lavage relies on multiple cut-off values which are all based on one single study [13], requiring further validation.

**Figure 1:**
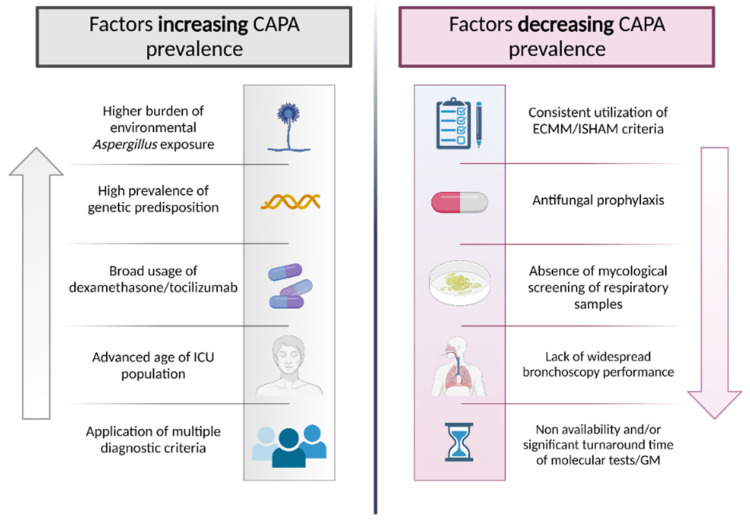
Factors increasing/decreasing prevalence of COVID-associated pulmonary aspergillosis (CAPA).

Even after the implementation of ECMM/ISHAM criteria, recently reported prevalence varies between centers [3,27]. From a present day perspective, these discrepancies could be explained by regional differences in therapeutic approaches to COVID-19ARF, alterations in diagnostic processes, included patient population, burden of fungal exposure, and host-specific predisposing genetic constitutions (Figure 1). Fungal defense mechanisms are mostly based on innate immune cells, including macrophages and polymorphonuclear neutrophils. These cells exhibit numerous pattern recognition receptors (PRRs), e.g., toll-like receptor (TLR) 4 or dectin-1, which are responsible for recognizing pathogen-associated molecular patterns (in the case of fungi, mainly cell wall components) and hence arbitrating further innate and adaptive immune responses. Aberrations in genes encoding for these PRRs were shown to significantly enhance susceptibility to IFIs, including invasive aspergillosis [31]. These genetically predisposing patterns are naturally subjected to demographic variances.

## 3. Strengths and Weaknesses of COVID-19 Autopsy Studies for Determining True CAPA Prevalence

Diagnosis of fungal disease is challenging, especially in the context if ICU. Critically ill patients may present several concomitant infectious and non-infectious processes that may mimic IFI. Accordingly, the only way to achieve a proven aspergillosis classification is to obtain histological diagnosis with evidence of invasive disease [32]. However, lung biopsies are rarely obtained in daily clinical practice and therefore autoptic studies are very important to ascertain the real pathogenetic role of *Aspergillus* or other fungal disease in this context, as happened in the past. As an example, autoptic studies were very helpful to understand the (poor) pathogenetic role of *Candida* spp. when isolated in lower respiratory tract of critically ill patients [33].

Post-mortem examination represents the most definitive method to detect invasive mold diseases, including CAPA. Autopsy studies are of particular value for CAPA for mainly two reasons: (1) Invasive pulmonary aspergillosis is generally accompanied by a low ante-mortem detection rate (~40%), and (2) previous autopsy studies suggested ante-mortem overdiagnosis particularly for respiratory tract infections [34,35]. When autopsy is performed with the intention to seek evidence for IFIs, presence of hyphae in histopathological investigation is simple to detect and hence a high likelihood of case identification is warranted. In terms of CAPA, Kula et al. did a systematic review of 50 autopsy studies from 15 different countries, including 677 decedents with severe COVID-19 infection with individual-level data for 443 [36]. Strengths of this review are the high number of investigated cases, performance of standard autopsy (in contrast to minimally invasive techniques) in 82% of decedents, availability (although not utilization) of fungal stain procedures in 91% of decedents, and declaration of important clinical information (e.g., mechanical ventilation status in 81% of decedents). An invasive mold disease was found in only 2% (11/677), with *Aspergillus* spp. representing the causative pathogen in eight cases, while in two cases mold identification was missing [36]. These findings provide a vigorous counterpart to the reported prevalence in clinical studies, indicating that angioinvasion may only occur in a proportion of cases with airway invasive disease.

Importantly, there are also weaknesses to consider when interpreting evidence from available autopsy studies. First of all, most studies included in the review of Kula et al. were not focused on diagnosis of IFI, because it was largely unknown as a relevant complication of COVID-19 ARF in the early phases of the pandemic when many of those studies were conducted [36]. Consistently, only 38% of autopsy examinations used routine fungal staining of lung tissue. Second, the rate of mechanical ventilated patients was 58%. Therefore, the studies were focused in part in patients with lower risk of CAPA according to recent observations. Third, the number of patients receiving anti-interleukin 6 or corticosteroids was on only 60 (<10%) which is very low in comparison with the current standard of treatment [37]. This may raise the question that some data on the index studies was underreported. Alternatively, this could be secondary to the period of publication of studies included in the review (all in 2020) [36]. In this initial phase of the pandemic, attention on CAPA was poor, and the armamentarium against COVID-19 was weak. It is likely that the prevalence of CAPA may have increased lately, because of the widespread use of immunomodulant therapies. Similarly the study included only few patients with immunocompromising conditions (6%) and this may have reduced the risk for CAPA. We believe that this population is likely to increase in the near future, because of lower response rate of antiSARS-CoV2 vaccine.

Surprisingly, other autopsy studies published later on, and were not included in the aforementioned systematic review, report different findings. In an Italian study reporting results of 45 consecutive autopsies of COVID-19 decedents, proven CAPA was diagnosed in 20% of cases. Of note, fungal staining was routinely performed in that study [38]. In another recent single center autopsy study, six cases of invasive mold diseases (CAPA N = 4, mucormycosis = 2) in eight decedents with severe COVID-19 infection were reported [39].

## 4. CAPA as an Important Opportunistic Infection in COVID-19 ICU Patients

Invasive pulmonary aspergillosis has been considered historically a problem in the immunocompromised host. However, in recent years, colonization or infection by *Aspergillus* spp. in the ICU has been increasingly reported. As an example, in the EPIC II study, an international multicenter study reporting the prevalence of infection in ICUs, reported a prevalence of positive culture of 1.4% in ICU patients with infection [40]. Conversely, a recent study reported a prevalence of 14% of probable pulmonary aspergillosis among ICU patients with ventilator-associated pneumonia [41]. The increased awareness, novel diagnostic methods and change in the population accessing intensive care may explain this wide difference. In COVID-19 ICU patients there are several additional factors that may allow to consider *Aspergillus* as an important opportunistic pathogen. From a pathophysiological standpoint, viral infections are considered an important trigger for fungal infection by the involvement of a number of immune pathways and mechanisms that may play a crucial role in the co-pathogenesis of viral and fungal lung infections. In fact it has been hypothesized that the damaged epithelium of the airway and suppression of cellular immunity, including defective antigen-specific cytotoxic T lymphocyte responses and impaired phagocyte activities such as phagocytosis, production of cytokines, and reactive oxygen species, formation of neutrophil extracellular traps and killing abilities, are the basis for viral and fungal co-infection. Both damaged epithelium and viral infection is likely to increase IFN production. In addition, IFN-α/β are also produced by alveolar macrophages. This hyperproduction of IFNs may, in turn, suppress monocyte, macrophage and neutrophil recruitment and effector responses that are essential against fungal infections [42].

In several studies assessing risk factors for CAPA among COVID-19 patients, most of attention was reserved for corticosteroids and immunomodulant agents. Corticosteroids are now considered as a backbone treatment for severe or critical COVID-19 infection and the large majority of ICU patients receive or have recently received dexamethasone. Steroids have potent, pleiotropic effects on the immune system that can predispose patients to developing life-threatening invasive aspergillosis. In several studies focused on settings different from COVID-19, corticosteroid use was associated with increased risk of pulmonary aspergillosis [43,44,45]. Studies focused on COVID-19 reported conflicting results. Several papers found that dexamethasone or other corticosteroids were associated with higher risk of CAPA [2,13,46], whereas others failed to find this association [27]. It has to be considered that, as corticosteroids represent a standard treatment for COVID-19, it could be possible that the lack of control without exposure may have biased the analysis in some cases. In a similar fashion, use of interleukin-6 blockers (mainly tocilizumab) was associated with CAPA [2,3].

## 5. Diagnosis and Antifungal Treatment of CAPA: How and When?

To date, the largest studies investigating epidemiology of CAPA uniformly report higher mortality rates in patients with CAPA (52%–71%) compared to patients without CAPA (32%–43%) [2,3,27], particularly when both BALF culture and BALF GM are positive [47]. Thus, a high level of alertness and clinical suspicion, optimally resulting in early diagnosis and initiation of antifungal treatment, is vital for sufficient management. Due to the unspecific clinical and radiological findings, mycological evidence represents a main component for establishing a diagnosis. Absence of neutropenia in patients with CAPA results in primarily airway invasive growth (in contrast to primarily angioinvasion in neutropenic patients) and broncho-alveolar lavage (BAL) samples are therefore the preferred sample type for CAPA diagnosis [4]. In contrast, GM from serum is exceedingly lacking sensitivity, indicating that it may only turn positive very late in the disease process once angioinvasion occurs. Recent studies have shown mortality rates of 80% and higher once serum GM or blood PCR become positive [1,26]. We summarized diagnostic performance of various tests based on recently published multicenter studies investigating CAPA cohorts (Table 1).

According to ECMM/ISHAM consensus criteria, various mycological criteria (i.e., BAL direct examination identifying *Aspergillus* hyphae, positive *Aspergillus* spp. culture in BAL, BAL GM of ≥1.0 optical density index (ODI), BAL qPCR of <36 Cq, and serum GM of >0.5) are each sufficient criteria to diagnose CAPA, without one being superior to another [32]. Combination of mycological criteria (e.g., qPCR + GM in BAL) may result in higher specificity [26]. In LMICs where bronchoscopy is scarcely available, validation of upper respiratory tract specimens (e.g., TA) is needed. Recent data suggest high sensitivity of the lateral flow assay (LFA)and GM enzyme linked immunosorbent assay (ELISA) from TA resulting in a high negative predictive value; TA GM testing could represent a potential tool for excluding CAPA, or at least rendering it much less likely. On the flipside, it is important to emphasize that TA GM testing lacks specificity [19,30]. Respiratory specimens, especially BAL for which common diagnostic tests and their respective cut-offs are validated, represent the cornerstone in CAPA diagnosis, in case bronchoscopy is available.

Current guidelines recommend treatment initiation as early as possible in order to hit during the airway invasive phase [32]. This may result in some overtreatment (i.e., treatment in patients that may not develop angioinvasive infection) and adverse drug events, yet there is no viable alternative approach, considering the high mortality rates in patients with CAPA in the absence of antifungal treatment, and those who receive antifungal treatment only once serum GM becomes positive [48]. Voriconazole or isavuconazole represent equal first line drugs, whereas liposomal amphotericin B (L-AMB) is the primary alternative. In suspected azole resistance, voriconazole/isavuconazole can be combined with an echinocandin and L-AMB as an equal alternative in this setting and first choice once resistance is proven [32]. Novel antifungals in the pipeline, namely fosmanogepix and olorofim, may have similar efficacy compared to azoles, yet without the same burden of limiting drug–drug interactions [49]. Hopefully they will overcome the bottleneck of limiting pharmacokinetics and toxicity of current azoles, becoming more favorable treatment options in the near future. While mold active antifungal prophylaxis has been shown to be successful in preventing CAPA in some single center cohort studies [50,51,52], larger level evidence is needed before this approach can be recommended.

## 6. Conclusions and Areas for Further Research

CAPA remains an important entity in the current pandemic context. While some of the earlier reported rates may have been overestimated in the absence of uniform consensus criteria in the early phases of the pandemic, utilization of more conservative uniform criteria has resulted in prevalence rates that are in median comparable between multicenter studies, although the rates may still vary widely between single centers. Despite the association between CAPA and mortality being consistently reported in the literature, several questions remain unanswered. Most importantly, a better diagnostic approach would be essential in guiding the clinician to a evidence based decision between early treatment or a “wait and see” strategy. As previously stated, treatment needs to be initiated early and could be useful in reducing the rate of angio-invasive disease and mortality but, on the other hand, very broad early (over) treatment could increase costs, adverse events, drug interactions and resistance. Realistic prevalence rates are important to avoid antifungal overtreatment, drug–drug interactions and drug-related adverse events in already highly vulnerable ICU patients [54]. Consistent application and constant refinement of diagnostic criteria is therefore pivotal in order to establish a realistic picture of CAPA frequency as implementation of therapeutic strategies are dependent and derive from these findings.

Novel approaches could be based on early treatment and, in the case of lack of confirmation, safe early withdrawal of antifungal therapy. Similarly, the optimal duration of treatment of antifungal for CAPA is yet to be established. Compared to recommendations in patients with prolonged neutropenia and invasive pulmonary aspergillosis, shorter duration of antifungal treatment for CAPA could be feasible, but studies are needed to evaluate when it is safe to stop antifungals. Finally, larger studies are needed to investigate a potential benefit of antifungal prophylaxis, particularly effects of inhaled antifungals on CAPA frequency and mortality. These studies might also be helpful to better understand the real role of CAPA as a driver of mortality in patients with severe COVID-19.

## Figures and Tables

**Table 1:** Diagnostic performance of various tests concluded from multicentric CAPA cohort studies [27,30,53].

Diagnostic Test/Study	Sensitivity	Specificity
BAL GM ODI > 1.0	74% (77/104)	99% (268/272)
BAL Culture	53% (56/106)	100% (298/298)
BAL LFA ODI > 1.0	52% (15/29)	98% (60/61)
BAL PCR	42% (48/115)	100% (49/49)
Serum GM ODI > 0.5	19% (20/106)	100% (379/380)
Serum BDG ≥ 80 pg/mL	38% (8/21)	85% (29/188)

BAL, bronchioalveolar lavage; BDG, β-d-glucan; GM, galactomannan; ODI, optical density index.

## Data Availability

Data available upon request.

## Abbreviations

ICU	Intensive Care Unit
ECMM/ISHAM	European Confederation of Medical Mycology/International Society for Human and Animal Mycoses
BAL	Bronchoalveolar Lavage
GM	Galactomannan
ODI	Optical Density Index
LFA	Lateral Flow Assay
BDG	β-D-Glucan
